# The prognosis of invasive micropapillary carcinoma compared with invasive ductal carcinoma in the breast: a meta-analysis

**DOI:** 10.1186/s12885-017-3855-7

**Published:** 2017-12-11

**Authors:** Yun Wu, Ning Zhang, Qifeng Yang

**Affiliations:** 1 Department of Breast Surgery, Qilu Hospital, Shandong University, No.107 West Wenhua Road, Jinan, Shandong 250012 People’s Republic of China; 2 Pathology Tissue Bank, Qilu Hospital, Shandong University, No.107 West Wenhua Road, Jinan, Shandong 250012 People’s Republic of China

**Keywords:** Invasive micropapillary carcinoma, IMPC, Breast cancer, Prognosis, Survival, Meta-analysis

## Abstract

**Background:**

Invasive micropapillary carcinoma (IMPC) of the breast is a rare variant of invasive ductal carcinoma (IDC). The prognosis of IMPC compared with that of IDC remains controversial; we conducted a meta-analysis to evaluate the prognostic difference between IMPC and IDC.

**Methods:**

We searched the PubMed, Cochrane Library, and EMBASE databases for relevant studies comparing overall survival (OS), disease-specific survival (DSS), relapse-free survival (RFS), local-regional recurrence-free survival (LRRFS) or distant metastasis-free survival (DMFS) rates between IMPC and IDC. Fixed-effect and random-effect models were utilized based on the heterogeneity of the eligible studies. Heterogeneity was further evaluated by subgroup and sensitivity analyses.

**Results:**

Fourteen studies with 1888 IMPC patients were included in the meta-analysis. The summarized odds ratio (OR) and 95% confidence interval (95% CI) was calculated to estimate the prognostic difference between IMPC and IDC. IMPC patients showed an unfavorable prognosis for RFS (OR; 2.04; 95% CI: 1.63–2.55) and LRRFS (OR: 2.82; 95% CI: 1.90–4.17) compared with IDC. However, no significant difference was observed in OS (OR: 0.93; 95% CI: 0.78–1.10), DSS (OR: 1.16; 95% CI: 0.95–1.40) and DMFS (OR: 0.95; 95% CI: 0.67–1.35) between IMPC and IDC. No obvious statistical heterogeneity was detected, except for DSS. Funnel plots and Egger’s tests did not reveal publication bias, except for RFS.

**Conclusions:**

This analysis showed that IMPC patients have a higher rate of loco-regional recurrence than IDC patients. However, OS, DSS and DMFS were not significantly different between IMPC and IDC. These results could help clinicians select therapeutic and follow-up strategies for IMPC patients.

**Electronic supplementary material:**

The online version of this article (10.1186/s12885-017-3855-7) contains supplementary material, which is available to authorized users.

## Background

Among females, breast cancer is the most common malignancy in both the developing and developed world, with over 1.3 million cases diagnosed and nearly 0.5 million deaths annually [1]. The 5-year survival rate of breast cancer was 89% from 2004 to 2010 in the United States, which significantly increased during the past 20 years due to early diagnosis through screening and the use of adjuvant systemic therapies [2, 3]. Different breast cancer subtypes show various prognoses, since breast cancer is essentially a heterogeneous disease. Breast cancer heterogeneity can be detected at different levels, and morphological heterogeneity manifests as different histologic types [4].

According to the most recent World Health Organization (WHO) classification, breast cancer can be classified in up to 21 distinct histological types on the basis of cell morphology, growth, and architecture patterns [5]. The most common histological type is invasive carcinoma of no special type, also known as invasive ductal carcinoma (IDC), which accounts for approximately 75% of all invasive breast cancers [5]. However, invasive micropapillary carcinoma (IMPC) was listed as a rare subtype of invasive breast carcinoma and constitutes between 2% and 8% of all breast cancers [5–12].

Invasive micropapillary carcinoma (IMPC) was first described in breast cancer as having an “exfoliative appearance” by Fisher et al. [13] in 1980, and first proposed as the term of “invasive micropapillary carcinoma” by Siriaunkgul et al. [14] in 1993. In clinical presentations, IMPC patients typically show a palpable mass with no obvious difference from IDC in radiographic findings, locations, and gross features [15]. Therefore, the microscopic evaluation of histological differences is vital to clinical diagnosis.

Compared with IDC, IMPC shows a more distinct morphologic architecture, characterized by pseudopapillary and tubuloalveolar arrangements of tumor cell clusters in sponge-like, clear empty spaces, thereby mimicking extensive lymphatic invasion [14]. The neoplastic cell displayed an “inside-out” pattern, known as reverse polarity, which could be observed by electron microscopy. Immunohistochemistry (IHC) for epithelial membrane antigen and sialyl Lewis X was applied to highlight the apical cellular membrane of peripheral cells at tumor nests and to confirm the diagnosis [16].

According to clinical experience, most patients with IMPC present extensive axillary lymph node (ALN) metastasis at initial diagnosis. Several studies of this subtype of breast cancer have demonstrated that IMPC has high proclivity to lymphovascular invasion (LVI) (35%–75%), ALN metastasis (44%–85%), local recurrence and distance metastasis [8, 10, 17, 18], which has gained increasing attention in the past 30 years. Due to highly lymphotropic nature and more aggressive behavior of IMPC, it was generally accepted that this disease shows unfavorable prognosis compared with IDC [10, 17]. Nevertheless, a number of recent studies have indicated that IMPC exhibited better or similar prognosis compared with IDC [19, 20]. For instance, Chen et al. [20] and Yu et al. [21] found that the overall survival (OS) was similar for IMPC and IDC patients; Chen et al. [20] also found that IMPC patients show a more favorable disease-specific survival (DSS) compared with IDC patients. However, Shi et al. [22] and Chen et al. [17] found that OS and relapse-free survival (RFS) were worse in the IMPC group than in the IDC group.

Because of the low incidence of this breast cancer variant and the relative scarce publications, there is some controversy about the prognosis of IMPC compared with IDC. The aim of current meta-analysis was to analyze the existing data to gain a clearer insight into the prognostic difference between IMPC and IDC. The results could help clinicians to determine counseling and follow-up strategies as well as tailor therapies to improve the outcomes of IMPC patients.

## Methods

### Publication selection

We searched PubMed and EMBASE databases and the Cochrane library in May 2017 for all studies reporting on IMPC and prognosis. Publications with the following search words in the title, abstract or key words were included: breast cancer, invasive carcinoma, micropapillary, invasive micropapillary carcinoma, prognosis, outcome and survival. Only studies written in English were included.

### Inclusion and exclusion criteria

The articles were included in the present analysis if they met the following criteria: (I) studies involved must compare the prognostic outcomes between IMPC and IDC; (II) detailed statistics should report outcomes, such as percentages of overall survival (OS), disease-specific survival (DSS), relapse-free survival (RFS), local-regional recurrence-free survival (LRRFS) or distant metastasis-free survival (DMFS); and (III) if the studied population was duplicated, only the previous publication or the publication with the largest sample size would be included. Publications meeting all three criteria would be included; otherwise, studies were excluded from this analysis to avoid the significant heterogeneity between studies and bias of this analysis. Reviews, meeting abstracts, letters, comments, editorials, and case reports were also excluded because of the limited data.

### Patterns of failure and survival analysis

We defined loco-regional recurrence (LRR) as the appearance of tumors in the ipsilateral breast, chest wall, ipsilateral axilla, internal mammary, supraclavicular area or infraclavicular area. Otherwise, recurrence was categorized as distant metastasis.

Overall survival (OS) and disease-specific survival (DSS) were measured from the data of surgery or diagnosis to the death and death due to breast cancer progression. Recurrence-free survival (RFS), LRR-free survival (LRRFS) and distant metastasis-free survival (DMFS) were measured from the date of operation to the date of LRR and/or distant metastasis, LRR and distant metastasis, respectively.

### Data extraction

The following information was extracted from each eligible publication: author names, year of publication, total number of IMPC and IDC patients, components of IMPC, TNM stage, duration of follow-up and survival data (percentages and number of events). When the essential information was not provided in the articles, every effort was made to contact the authors.

### Statistical analysis

The summarized statistics of odd ratios (ORs) and 95% confidence intervals (CIs) were calculated using a fixed-effect model when there was minimal heterogeneity among studies to assess the correlation between IMPC and prognosis. The χ^2^ and Ι^2^ test methods were utilized to determine the heterogeneity across studies of the ORs. When *p* < 0.05 and/or Ι^2^ > 50% which indicate significant heterogeneity, the random-effect model was used. Reasons for statistical heterogeneity were explored through subgroup or sensitivity analyses (when Ι^2^ > 50%). Each publication was weighted according to the size of the sample. Forest plots were generated using standard techniques to pool the included studies, for which horizontal lines signify 95% CI, the area of each square represents the weight, and the position of each square demonstrates the OR estimate. The vertical line at the null value showed that OR was 1.0. The overall summarized prognostic estimate under fixed effects with its CI was shown.

The potential publication bias was evaluated by the funnel plot and Egger’s tests. An asymmetrical plot suggests potential publication bias. Funnel plot asymmetry was assessed by Egger’s linear regression test, which was applied to measure funnel plot asymmetry on the natural logarithm scale of the OR. The significance of the intercept was determined by the t-test according to Egger (*p* < 0.05 was considered representative of statistically significant publication bias). All *p* values were two-sided, and p < 0.05 was considered as statistically significant. All statistical analyses were performed with STATA software (version 12.0; Stata Corporation, College Station, TX, USA).

## Results

### Eligible studies

**Fig. 1:**
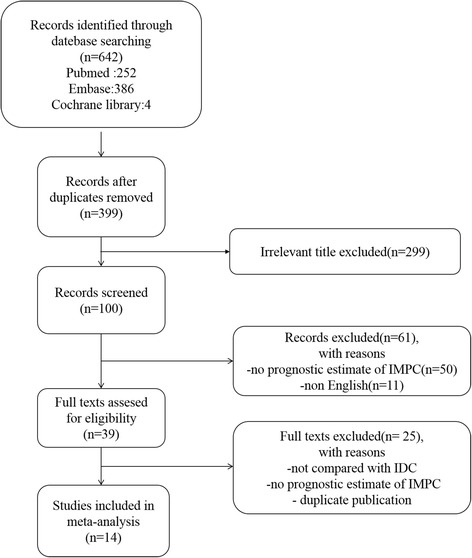
Flow diagram illustrating the selection of the included studies

The search yielded 642 publications. After removing the duplicates, the titles of the remaining publications were evaluated, and 299 publications were initially excluded. The abstracts of the publications 100 records were screened, and 61 publications were excluded. Thirty-nine publications were potential eligible studies that mentioned identifying the correlation of IMPC and prognosis. Twenty-five studies were excluded for not providing adequate, detailed statistics or not comparing with IDC. Thus, based on the criteria described above, 14 publications were eligible for inclusion in this meta-analysis [12, 17, 19–30]. The search strategy and filters applied are shown in Fig. 1.

### Study characteristics

Ultimately, a total of 1888 IMPC patients from the 14 publications, which had survival data, were included in this meta-analysis. The characteristics of these studies are detailed in Table 1. Most (*n* = 13) of the studies recruited patients with all subtype IDC patients as the control group, but one study focused on the triple negative IDC [24]. In clinical practice, the term “pure IMPC” is defined as IDC constituting at least 75% of the micropapillary component [16]. The patients in two studies were pure IMPC patients, the patients in eleven studies were mixed patients, and the patients in one study were unknown.

**Table 1:** Characteristics of eligible studies

Study	Study period	No. of cases		Component of IMPC*	TNM stage	Follow-up time(M)	Survival data ^a^				
		IMPC	IDC				OS	DSS	RFS	LRRFS	DMFS
Tang [30]	2017	170	728	mixed	I,II,III	median 40	94.5% vs 90.6% (*P* = 0.592)	–	82.1% vs 90.6% (*P* = 0.001)	86.9% vs 97.1% (P < 0.001)	91.5% vs 88.3% (*P* = 0.923)
Li [23]	2016	33	347	mixed	I,II,III	median 39	97% vs 94.2% (*P* = 0.78)	–	87.9% vs 86.2% (*P* = 0.88)	93.9% vs 89.0% (P = 0.88)	90.9% vs 89.0% (*P* = 0.97)
Yu [21]	2015	267	267	mixed	I,II,III	median 59	97.7% vs 95.7% (*P* = 0.67)	–	85.0% vs 92.1% (*P* = 0.07)	91.8% vs 96.3% (*P* = 0.03)	91.4% vs 93.3% (*P* = 0.52)
Chen [24]	2015	95	200	mixed	I,II,III	median 60	81.9% vs 79.8% (*P* = 0.475)	–	–	71.4% vs 89.8% (*P* < 0.001)	79.8% vs 73.7% (*P* = 0.091)
Shi [22]	2014	188	1289	mixed	I,II,III	median40.5	–	67.1% vs 87.5% (P < 0.001)	75.9% vs 89.5% (P < 0.001)	–	–
Liu [19]	2014	51	102	mixed	I,II,III	median 51	–	–	84.3% vs 78.4% (*P* = 0.606)	–	–
Chen [20]	2014	636	297,735	unknown	I,II,III,IV	median 48	82.9% vs 80.5% (*P* = 0.086)	91.8% vs 88.6% (P = 0.086)	–	–	–
Vingiani [25]	2013	49	13,487	pure	I,II,III	median 51	89.8% vs 90.8% (*P* = 0.80)	–	75.5% vs 79.6% (*P* = 0.48)	–	–
Gokce [12]	2013	103	34	mixed	I,II,III	mean 63.5	–	75.9% vs 82.4% (*P* = 0.44)	–	–	–
Kuba [26]	2011	10	162	mixed	I,II,III	median 72	–	90% vs 94.4%	90% vs 89.8%	–	–
Yu [27]	2010	72	144	mixed	I,II,III	median 45	86.0%vs 87.7% (*P* = 0.18)	–	68.2% vs 81.4% (*P* = 0.046)	84.7% vs 94.4% (*P* = 0.0024)	78.1% vs 79.3% (*P* = 0.87)
Kim [28]	2010	61	221	pure	I,II,III	mean 38.6	–	–	86.9% vs 94.6% (*P* = 0.049)	–	–
Chen [17]	2008	100	100	mixed	I,II,III,IV	mean 60.1	59% vs 77% (*P* = 0.004)	63.3% vs 81% (*P* = 0.005)	–	–	–
Zekioglu [29]	2004	53	60	mixed	I,II,III	mean 56.5	–	72.2% vs 81.7% NS	–	–	–

Abbreviations: IMPC, Invasive micropapillary carcinoma; IDC, Invasive ductal carcinoma; OS, Overall survival; DSS, Disease-specific survival; RFS, Relapse-free Survival; LRRFS, Local-regional recurrence free survival; DMFS, Distant metastasis-free survival; NS, Not significant

^a^All percentage was for IMPC vs IDC%

*At least 75% of the micropapillary component identified in an IDC to be defined as pure IMPC

### Survival outcomes of IMPC and IDC

**Fig. 2:**
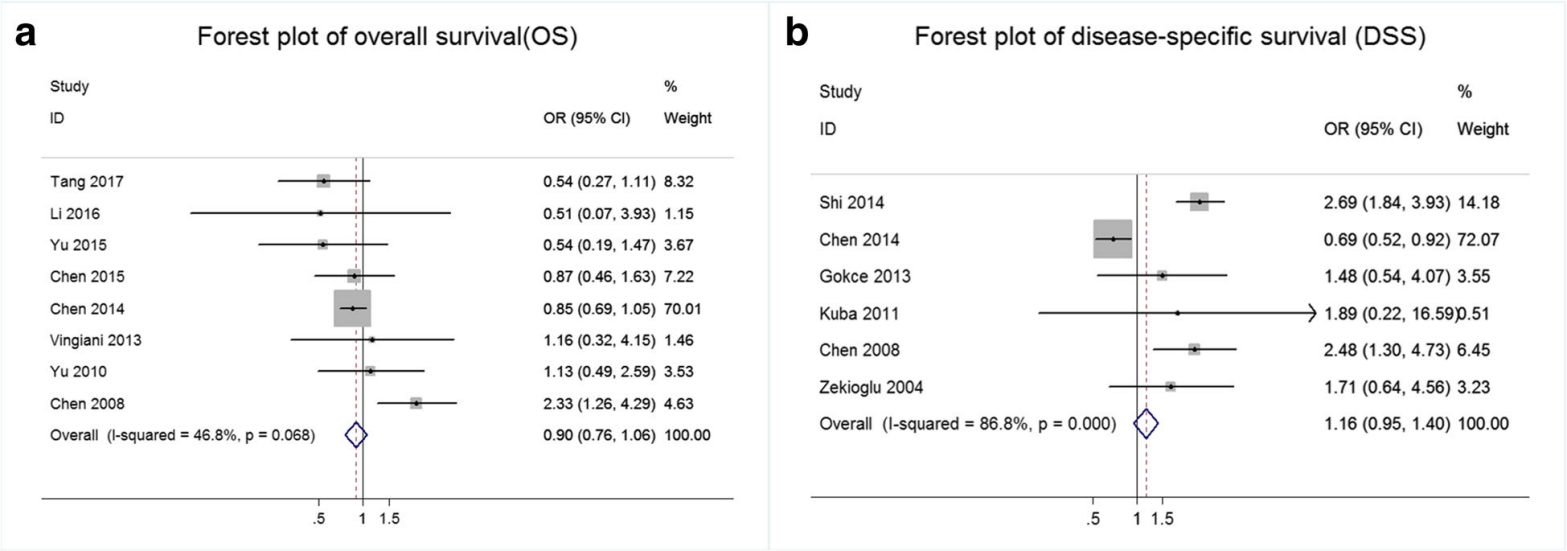
Results of the survival analysis in IMPC compared with IDC. **a** Forest plot of the odds ratio (OR) for overall survival (OS) from eligible studies. **b** Forest plot of the odds ratio (OR) for disease-specific survival (DSS) from eligible studies

Only 8 of the 14 studies provided OS data (*n* = 301,041 patients). The ORs and 95% CIs for each study and the summarized OR are shown in Fig. 2a. The individual OR of the 8 articles ranged from 0.51 to 2.33. The overall summarized estimate OR was 0.90 (95% CI: 0.76–1.06). There was no significant heterogeneity across the studies (Ι^2^ = 46.8%, χ^2^ = 13.15, *p* = 0.068). Using the random-effects method yielded a similar effect estimate (OR = 0.94, 95% CI: 0.67–1.32).

Seven studies (*n* = 300,528 patients) provided DSS data. The ORs and 95% CIs for each study and the summarized OR are shown in Fig. 2b. The OR from each of the 6 studies ranged from 0.69 to 2.69. The overall summarized estimate OR was 1.16 (95% CI: 0.95–1.40), with a higher heterogeneity (Ι^2^ = 84.5%, χ^2^ = 38.63, *p* < 0.001).

**Fig. 3:**
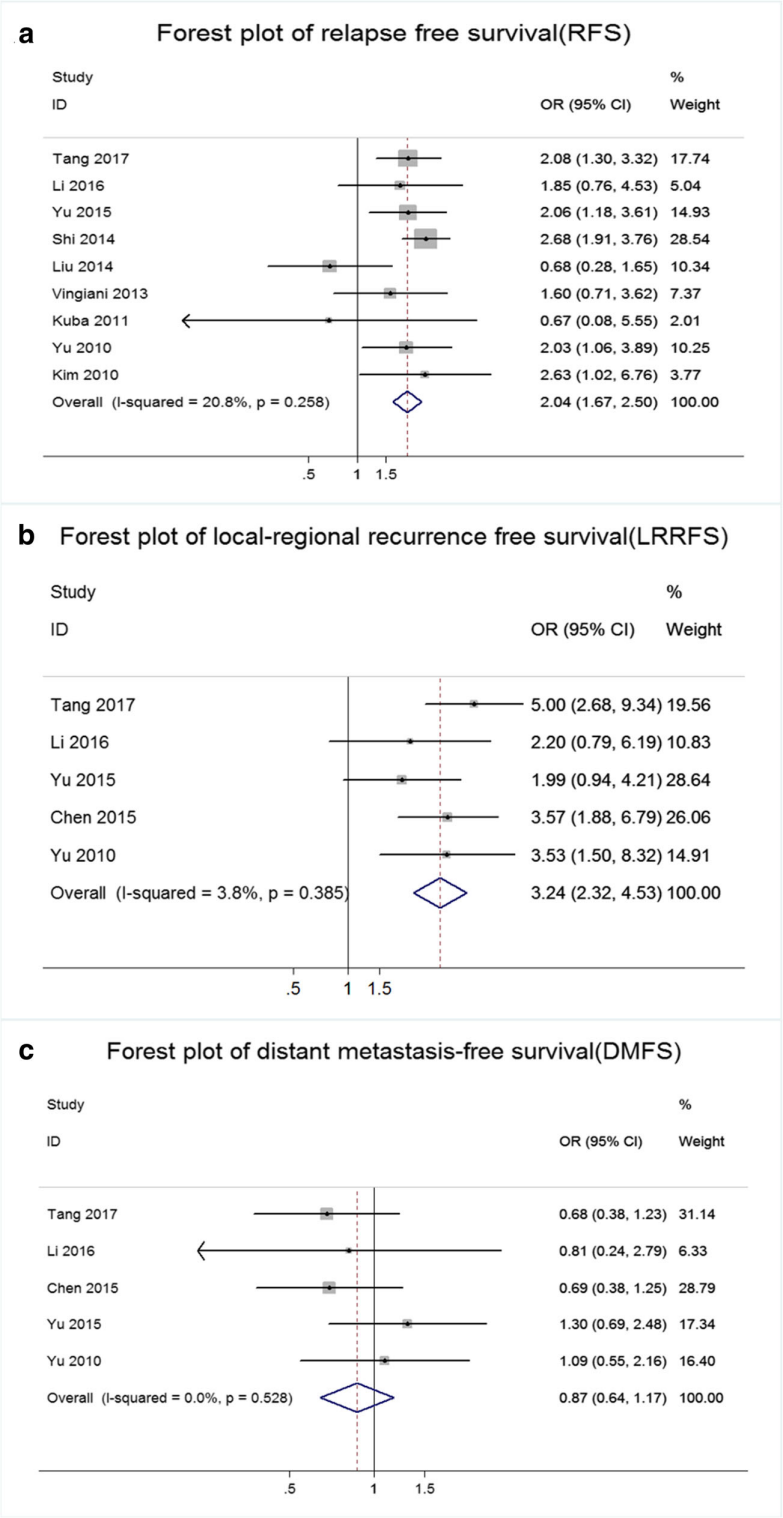
Results of the recurrence analysis in IMPC compared with IDC. **a** Forest plot of the odds ratio (OR) for relapse-free survival (RFS) from eligible studies. **b** Forest plot of the odds ratio (OR) for local-regional recurrence-free survival (LRRFS) from eligible studies. **c** Forest plot of the odds ratio (OR) for distant metastasis-free survival (DMFS) from eligible studies

Nine articles (*n* = 4259 cases) provided RFS data. The OR and 95% CI for each study and the summarized OR are shown in Fig. 3a. The ORs of the 8 studies ranged from 0.67 to 2.68. The overall summary estimate OR was 2.04 (95% CI: 1.67–2.50), with no significant evidence of heterogeneity (Ι^2^ = 20.8%, χ^2^ = 10.10, *p* = 0.27).

Five studies (*n* = 2323 patients) provided LRRFS data. The OR and 95% CI for each study and the summarized OR are shown in Fig. 3b. The OR from each of the 4 studies ranged from 1.99 to 5.01. The overall summarized estimate OR was 3.24 (95% CI: 2.32–4.53). Heterogeneity testing revealed Ι^2^ = 3.8% and χ^2^ = 4.16, *p* = 0.39.

Five studies (n = 2323 patients) provided DMFS data. The OR and 95% CI for each study and the summary OR are shown in Fig. 3c. The OR from each of the 4 studies ranged from 0.69 to 1.30. The overall summarized estimate OR was 0.87 (95% CI: 0.64–1.17). There was also no significant heterogeneity across the studies (Ι^2^ = 0.0%, χ^2^ = 3.18, *p* = 0.53).

### Publication bias

Funnel plots were generated to detect potential publication bias (Additional file 1: Figure S1 and Additional file 2: Figure S2), which did not reveal remarkable asymmetry. Egger’s tests were also performed to detect publication bias to diminish the subjective bias. No evident publication bias was detected by Egger’s tests for survival data, except for RFS. The *p*-values for Egger’s test of OS, DSS, RFS, LRRFS and DMFS were 0.89, 0.44, 0.04, 0.271 and 0.83, respectively.

## Discussion

Breast cancer is the most common cancer in women and the primary cause of death among women globally [2]. Breast cancer heterogeneity can be detected at different levels, from the classic histopathological characterization to the more modern molecular classification. According to the WHO, breast cancer can be classified into 21 distinct histological types, which include non-invasive breast cancer, including ductal carcinoma in situ, lobular carcinoma in situ and Paget’s disease and invasive breast cancers, such as invasive ductal carcinoma and some rare cancer types [5].

IMPC of the breast is a morphologically distinct subtype of breast carcinoma, associated with a highly lymphotropic nature and aggressive clinicopathological features and tends to have a higher clinical stage at initial diagnosis in IMPC patients [20]. IDC is the most common type of breast cancer, accounting for up to 75% of invasive breast carcinomas [15]. In clinical practice, the histopathological classification has a prognostic value. Clinicians typically presumed that a more histopathologically aggressive carcinoma may indicate a worse prognosis. However, there remains discordance in the published literatures regarding whether IMPC patients show a worse prognostic outcome than IDC patients.

Numerous reports have been published in the past three decades to compare the prognostic difference between IMPC and IDC [12, 17, 19–30]. Chen et al. [20], using the US National Cancer Institute’s Surveillance, Epidemiology, and End Results (SEER) database, found that the overall prognostic results of IMPC were similar to those of IDC and showed a more favorable DSS than IDC patients, although lymph node metastasis was more common in the former. Yu et al. [21] demonstrated a significantly worse LRRFS and RFS for IMPC compared with matched IDC controls, however no difference was observed in the overall survival analysis. In contrast, Shi et al. [22] and Chen et al. [17] showed that OS and RFS were poorer in the IMPC group than in the IDC group. However, since the incidence of IMPC was low, the majority of studies only reported on small sample sizes with limited follow-ups. The present meta-analysis summarized the current evidence of the prognostic difference between IMPC and IDC. Based on the combined data of 14 eligible published studies, we observed that IMPC is an unfavorable prognostic factor for RFS (*p* < 0.001) and LRRFS (p < 0.001), while no significant difference was detected in OS (*p* = 0.20), DSS (*p* = 0.14) and DMFS (*p* = 0.35) between IMPC and IDC.

Eight of the fourteen studies referred to the OS, and only Chen et al. [17] suggested that the OS of IMPC is comparable to that of IDC. Furthermore, we observed that IMPC showed unfavorable RFS and LRRFS due to its lymphotropic nature and aggressive behavior. However, we revealed a similar DMFS compared with IDC since the aggressive behavior of IMPC mainly embodied a higher rate of lymph node involvement rather than distant metastasis, in contrast to another aggressive subtype, triple negative IDC [24]. We suspected that the treatment therapy may explain the contradiction between unfavorable local relapse and better overall survival. According to the guidelines of the National Comprehensive Cancer Network, the lymphatic involvement of breast cancer will always lead to radical surgical excision as well as post-mastectomy radiotherapy for patients with more than 3 involved nodes, which will contribute to the overall survival of these patients. Considering the higher rate of loco-regional recurrence in IMPC patients, we recommend that clinicians adopt sufficient local treatments, such as regional surgical resection and local radiotherapy. Moreover, clinicians should pay more attention to the loco-regional recurrence for IMPC patients at follow-up.

Two of the fourteen studies included pure IMPC patients, while eleven studies included mixed patients and the patients in one study were unknown. Most of the included patients are of mixed histological type. According to previous studies, IMPC may occur either alone or in combination with other histological types of breast cancer, such as IDC, mucinous carcinoma, and ductal carcinoma in situ [7, 24, 31], and most patients had mixed IMPC [12, 22]. The prognosis might potentially be affected by other histological components. However, Chen et al. [17] showed that tumors containing less than a 25% IMPC components were still associated with a significantly higher incidence of LVI and LN metastasis than IDC. Ide et al. [11] observed that the presence of the IMPC component alone was a significant predictive factor for LN metastasis, even if it was detected in only a small proportion of the tumor. Marchio et al. [32, 33] demonstrated that in histological features and molecular genetic profiles, mixed IMPC patients were more closely related to pure IMPC patients than to IDC patients. Thus, we suspected that the presence of the IMPC component correlates with the aggressive behavior of the tumor, regardless of the IMPC component proportion.

Additionally, we did not observe any obvious statistical heterogeneity among the studies included in the present analysis, except for DSS. Thus, we performed subgroup and sensitivity analyses to explore the heterogeneity. Subgroup analysis revealed that race potentially caused heterogeneity (Additional file 3: Figure S3). We observed that Asian IMPC patients showed an unfavorable DSS compared with Asian IDC patients (OR = 2.61, 95% CI = 1.88–3.61). However, IMPC was a favorable prognostic factor in Caucasian populations (OR = 0.77, 95% CI = 0.60–0.99). These results may be affected by different genetic backgrounds and socioeconomic factors that may influence a woman’s adherence to recommendations for clinical breast examination and the likelihood of the woman to seek appropriate care when a breast mass is detected [34–36]. Thus, these factors may be related to the degree of lymph node positivity at initial diagnosis, which was a significant prognostic factor in IMPC patients. Chen et al. showed lower lymph node positivity (52%) than the range (68.8–90.5%) reported in other large studies of IMPC [6, 7, 17, 20, 27, 29, 37–39], likely accounting for the favorable prognosis of DSS in Caucasian populations. These results emphasize the importance of early diagnosis for IMPC patients. To evaluate the influence of a single study on the pooled OR, we performed a sensitivity analysis by estimating the average OR in the absence of each study. The results indicated that Chen et al. [20] influenced the OR of DSS. Chen et al. [20] concluded that IMPC showed a more favorable DSS than IDC, in contrast to the general understanding, thereby influencing the DSS heterogeneity. Chen et al. [20] speculated that higher ER positivity and a decreased rate of lymph node metastasis resulted in a favorable DSS outcome. Compared with other eligible studies, the most significant differences were TNM stage and the rate of lymph node positivity of IMPC patients, which may influence the eventual outcome. The lower lymph node positivity, lower TNM stage and notably slightly higher mastectomy rate of IMPC patients from Chen et al. [20] may suggest that these patients received radical therapy in the initial stage of the disease, therefore showing a favorable DSS rate of IMPC patients.

Among all of the studies included in the present analysis, we did not detect significant statistical publication bias. However, Egger’s test for RFS showed an obvious publication bias (*p* = 0.04), which may influence the robustness of the result of RFS. Thus, it cannot be determined whether the IMPC patients show a worse RFS than IDC patients. Notably, some studies that yielded negative results might not have been published at all, further increasing the potential influence of publication bias. The funnel plot generated to investigate this potential influence did not reveal any obvious asymmetry, and hence publication bias was not apparent.

Moreover, our meta-analysis has several limitations requiring consideration. First, the number of available, eligible publications is relatively small and may contribute to potential publication bias. Only scattered studies provided information concerning LRRFS and DMFS in the IMPC and IDC patients. Second, important pathological parameters, such as grade, molecular type (her2 status, hormone receptors), components of IMPC and administered therapies, such as chemotherapy, radiotherapy, etc., which may play a prognostic role and affect management decisions, were not consistently reported across all studies and was not considered in the present meta-analysis. Third, there were some notable clinical differences among the eligible studies (i.e., the criteria of eligible patients, component of IMPC, TNM stages, molecular subtypes of the patients included, median age and duration of follow-up) requiring further discussion. Finally, the retrospective design of the studies included in the present meta-analysis limits its power. Future prospective trials are needed to validate these results.

## Conclusions

The present study is the first meta-analysis to explore the prognostic differences between IMPC and IDC. The results suggested that IMPC patients have a higher rate of loco-regional recurrence than IDC patients, although no obvious difference in OS, DSS and DMFS compared with IDC was detected. More large-scale retrospective studies or prospective clinical trials of IMPC are needed to contribute to individualized and tailored therapy, which may improve the clinical management and outcomes of IMPC.

## Additional files

Additional file 1: Figure S1. (A) Funnel plot to detect publication bias for overall survival (OS). (B) Funnel plot to detect publication bias for disease-specific survival (DSS). (TIFF 304 kb)

Additional file 2: Figure S2. (A) Funnel plot to detect publication bias for relapse-free survival (RFS). (B) Funnel plot to detect publication bias for local-regional recurrence-free survival (LRRFS). (C) Funnel plot to detect publication bias for distant metastasis-free survival (DMFS). (TIFF 261 kb)

Additional file 3: Figure S3. Subgroup analysis of DSS according to different ethnicities. (TIFF 1627 kb)

## Abbreviations

95% CI: 95% confidence interval
ALN: Axillary lymph node
DMFS: Distant metastasis-free survival
DSS: Disease-specific survival
IDC: Invasive ductal carcinoma
IMPC: Invasive micropapillary carcinoma
LRRFS: Local-regional recurrence free survival
LVI: Lymphovascular invasion
OR: Odds ratio
OS: Overall survival
RFS: Relapse-free Survival
