# Supplementary figures and images for: The prognosis of invasive micropapillary carcinoma compared with invasive ductal carcinoma in the breast: a meta-analysis
Source: BMC Cancer. 2017 Dec 11;17:839. doi: 10.1186/s12885-017-3855-7 (PMC5725780; doi:10.1186/s12885-017-3855-7)

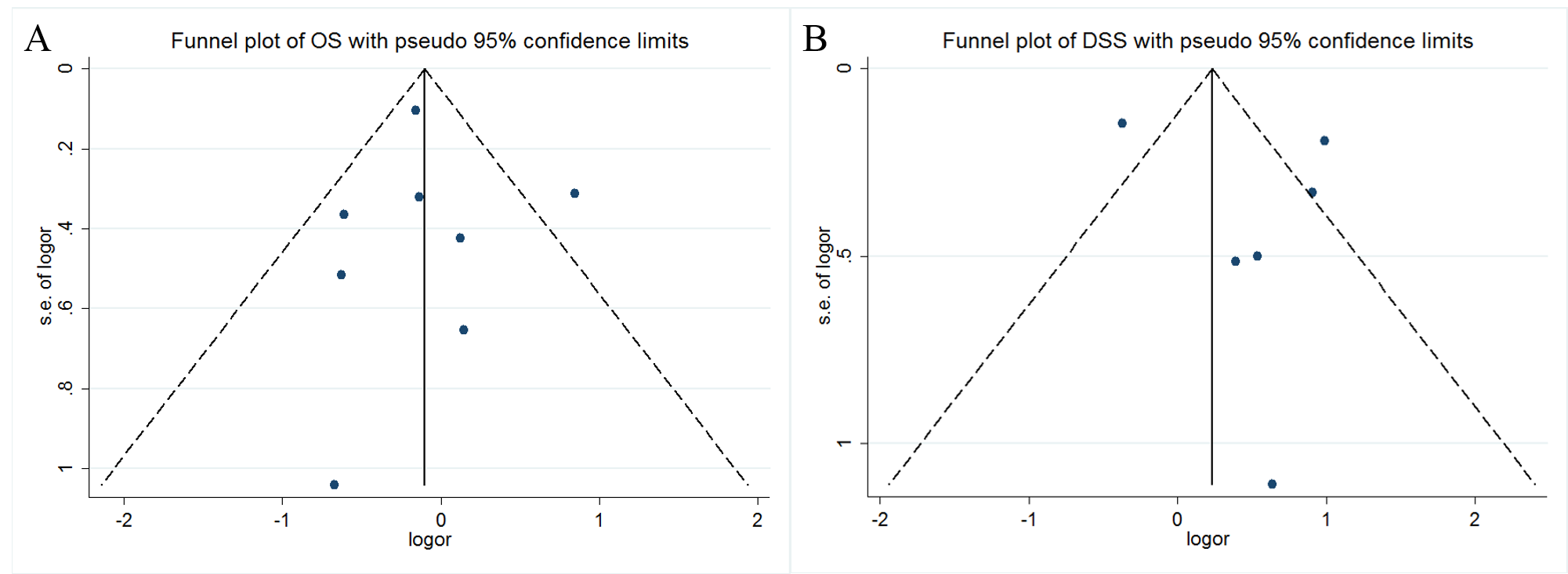

Supplement: Supplementary file 1 — (A) Funnel plot to detect publication bias for overall survival (OS). (B) Funnel plot to detect publication bias for disease-specific survival (DSS). (TIFF 304 kb) [file 12885_2017_3855_MOESM1_ESM.tif]

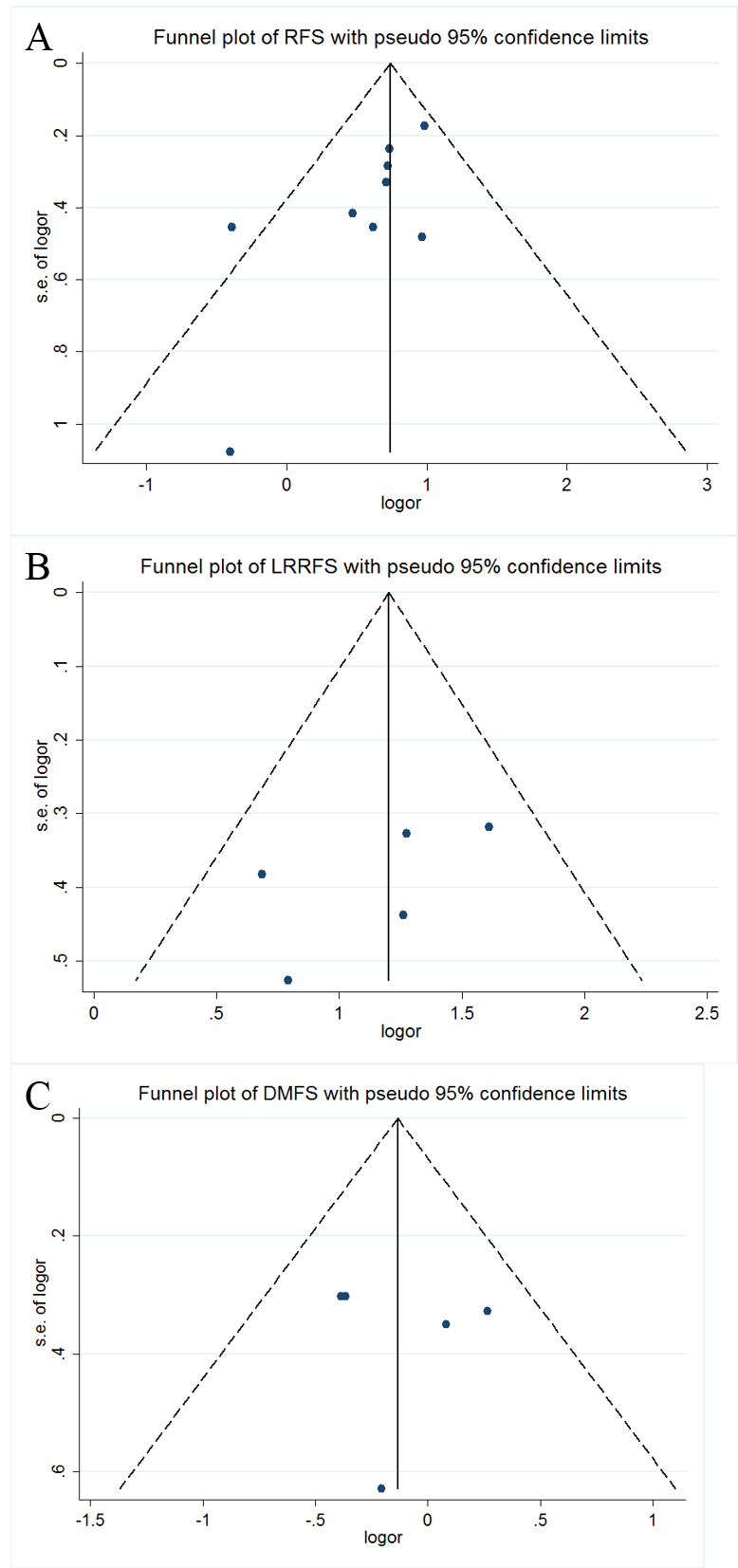

Supplement: Supplementary file 2 — (A) Funnel plot to detect publication bias for relapse-free survival (RFS). (B) Funnel plot to detect publication bias for local-regional recurrence-free survival (LRRFS). (C) Funnel plot to detect publication bias for distant metastasis-free survival (DMFS). (TIFF 261 kb) [file 12885_2017_3855_MOESM2_ESM.tif]

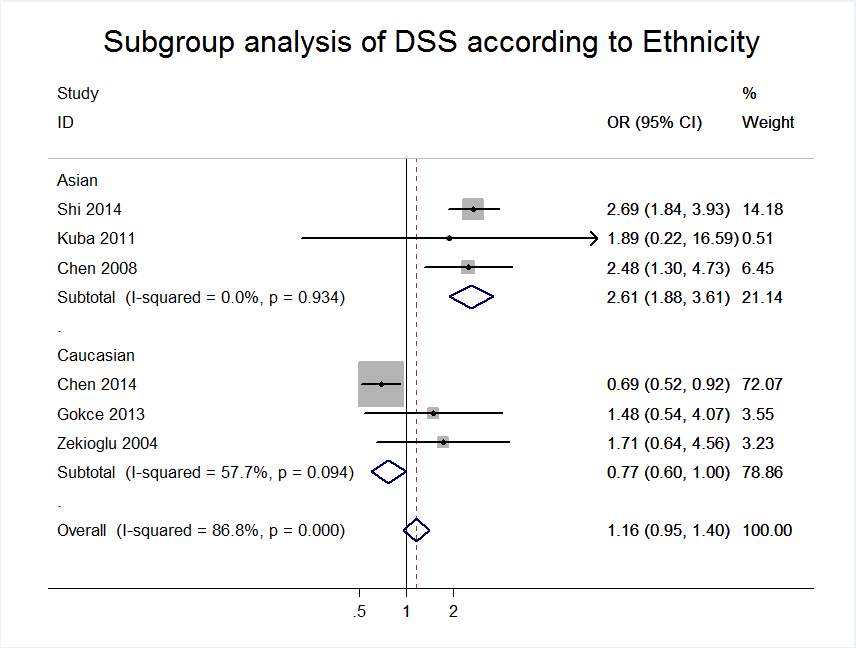

Supplement: Supplementary file 3 — Subgroup analysis of DSS according to different ethnicities. (TIFF 1627 kb) [file 12885_2017_3855_MOESM3_ESM.tif]
